# Innovative developments and emerging technologies in RNA therapeutics

**DOI:** 10.1080/15476286.2022.2027150

**Published:** 2022-02-19

**Authors:** François Halloy, Annabelle Biscans, Katherine E. Bujold, Alexandre Debacker, Alyssa C. Hill, Aurélie Lacroix, Olivia Luige, Roger Strömberg, Linda Sundstrom, Jörg Vogel, Alice Ghidini

**Affiliations:** a Department of Paediatrics, Medical Sciences Division, University of Oxford, Oxford, UK; b Oligonucleotide Chemistry, Discovery Sciences, BioPharmaceuticals R&d, AstraZeneca, Gothenburg, Sweden; c Department of Chemistry & Chemical Biology, McMaster University, (Ontario), Canada; d MiNA Therapeutics, Translation & Innovation Hub, London, UK; e Institute of Pharmaceutical Sciences, Department of Chemistry and Applied Biosciences, Eth Zürich, Zürich, Switzerland; f Sixfold Bioscience, Translation & Innovation Hub, London, UK; g Department of Biosciences and Nutrition, Karolinska Institutet, Sweden; h Mechanistic and Structural Biology, Discovery Sciences, BioPharmaceuticals R&d, AstraZeneca, Gothenburg, Sweden; i Helmholtz Institute for RNA-based Infection Research (Hiri), Helmholtz Center for Infection Research (Hzi), Würzburg, Germany; j RNA Biology Group, Institute for Molecular Infection Biology, University of Würzburg, Würzburg, Germany

**Keywords:** RNA-therapeutics, antisense, siRNA, delivery, saRNA, nucleic acid technologies, viral RNA, oligonucleotide based artificial ribonucleases, PROTAC

## Abstract

RNA-based therapeutics are emerging as a powerful platform for the treatment of multiple diseases. Currently, the two main categories of nucleic acid therapeutics, antisense oligonucleotides and small interfering RNAs (siRNAs), achieve their therapeutic effect through either gene silencing, splicing modulation or microRNA binding, giving rise to versatile options to target pathogenic gene expression patterns. Moreover, ongoing research seeks to expand the scope of RNA-based drugs to include more complex nucleic acid templates, such as messenger RNA, as exemplified by the first approved mRNA-based vaccine in 2020. The increasing number of approved sequences and ongoing clinical trials has attracted considerable interest in the chemical development of oligonucleotides and nucleic acids as drugs, especially since the FDA approval of the first siRNA drug in 2018. As a result, a variety of innovative approaches is emerging, highlighting the potential of RNA as one of the most prominent therapeutic tools in the drug design and development pipeline. This review seeks to provide a comprehensive summary of current efforts in academia and industry aimed at fully realizing the potential of RNA-based therapeutics. Towards this, we introduce established and emerging RNA-based technologies, with a focus on their potential as biosensors and therapeutics. We then describe their mechanisms of action and their application in different disease contexts, along with the strengths and limitations of each strategy. Since the nucleic acid toolbox is rapidly expanding, we also introduce RNA minimal architectures, RNA/protein cleavers and viral RNA as promising modalities for new therapeutics and discuss future directions for the field.

## Introduction

The use of a synthetic oligonucleotide to alter protein expression through Watson–Crick hybridization of RNA was first described by Zamecnik and Stephenson in 1978 [1]. From this discovery, it took 20 years of research in nucleic acid medicinal chemistry to fully establish synthetic nucleic acids as effective RNA-targeting drugs (RTDs). These have now become a very active field of drug design and development in the pharmaceutical industry and a total of 14 RTDs have been approved to date, with several more in clinical trials [2,3]. Currently, marketed RTDs are either single-stranded antisense oligonucleotides (ASOs) or double-stranded short interfering RNAs (siRNAs) engaging a messenger RNA (mRNA) target through Watson–Crick base pairing. However, nucleic acids in their native state are highly sensitive to nuclease degradation and show poor cellular uptake. As a result, RTDs on the market all incorporate chemical modifications at the backbone, base, or sugar level to improve their biological stability and optimize their pharmacokinetics properties. Consequently, current endeavours in RTDs seek to (i) improve their medicinal chemistry, (ii) achieve functional delivery beyond the liver, and (iii) increase cytoplasmic delivery through endosomal escape, in order to increase the amount of RTD available for activity. Despite these challenges, the performance of RTDs in human therapy and the broad scope of modifications available to date have fuelled the development of new modalities independent from classical antisense and RNA interference (RNAi) mechanisms, which are now the subject of several drug discovery projects in both industrial and academic research. In this survey, we provide a contemporary overview of major emerging nucleic acid-based technologies. We start with the most recent findings in the mature siRNA and antisense oligonucleotide fields. We then introduce promising RTD modalities under development in academic and commercial projects: antibiotic ASOs, viral prohead RNAs, small-activating RNAs, nucleic acid nanostructures, nucleic acid ribonucleases, nucleic acid – proteolysis targeting chimeras (PROTACs), and artificial ribonucleases (OBANs). The intent is to highlight the possibility of employing the extensive knowledge and progress in nucleic acids technology to develop sophisticated drugs and fully realize their potential as therapeutics.

## I. Innovative developments in nucleic acid therapeutics

### Overview of current single-stranded nucleic acid therapeutics and scope

Single-stranded oligonucleotides (ssONs) encompass three main groups of therapeutics: RNase-H active oligonucleotides, steric-blocking oligonucleotides, and aptamers. RNase-H active and steric-blocking oligonucleotides that bind an RNA target in a sequence-specific manner have been dubbed antisense oligonucleotides (ASOs). On one hand, RNase-H active oligonucleotides bind a cognate pre-mRNA or mature mRNA sequence, which triggers RNase-H binding and cleavage of the mRNA target [4]. This mechanism has been exploited for protein knockdown and also for the knockdown of pathogenic RNA species, such as those found in repeat-expansion diseases [5]. On the other hand, steric-blocking oligonucleotides do not degrade their mRNA target but, rather, mask a cognate sequence upon hybridization and prevent further binding by RNA-binding proteins and intracellular RNA species, thus blocking protein translation. A sub-category of steric-blocking oligonucleotides have been exploited for the silencing of a class of intracellular, non-coding RNAs called microRNAs (miRNAs), which bind mRNA transcripts and exert a regulatory role [6]. These oligonucleotides are dubbed anti-microRNAs (antimiRs). Another firmly established use of steric-blocking oligonucleotides is in splicing modulation with splice-switching oligonucleotides (SSOs). SSOs are designed to bind pre-mRNAs in the nucleus and modulate their maturation by masking splicing regulatory elements or splice sites [7,8]. Finally, aptamers are a third class of ssONs. They bind a given protein or receptor and modulate its function for a therapeutic outcome [9]. Aptamers are typically identified after successive enrichment rounds from a random pool of sequences leading to oligonucleotide sequences that bind their target with high affinity (e.g. SELEX procedure). With this wide variety of therapeutic modalities, ASO research is now focused on optimizing their therapeutic effects *in vivo* and expanding the scope of disease targets that can be effectively addressed. Current ASO research can be broken down into three aims: progressing on ASO medicinal chemistry; enhancing tissue-specific targeting and delivery; and gaining a better understanding of ASO internalization into cells and endocytosis.

### Challenges and strategies in the delivery of single-stranded antisense oligonucleotides

**Figure 1.**
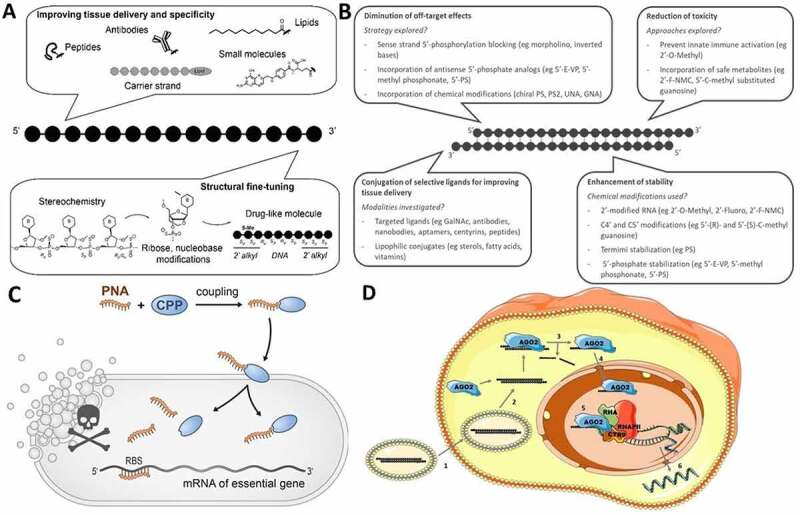
(A) Challenges and strategies for developing efficient and safe ssONs in the clinic. (B) Challenges and strategies for the development of efficient and safe siRNAs. (C) RNA-centric killing of a bacterium of interest can be achieved by delivering a short antisense oligomer (ASO), here a peptide nucleic acid (PNA), to sequester the 5’ region of the mRNA of an essential gene. Such ASOs are coupled to small uptake or cell-penetrating peptides that carry them inside the bacteria. The mechanisms of transport into the bacteria and whether peptide and ASO remain attached to each other, or cleaved after entry, are not completely understood. (D) Suggested mechanism for small activating RNAs. 1) The double stranded saRNA is taken up into the cell by endocytosis. 2) Then, the double stranded saRNA is loaded into an AGO2 protein. 3) The passenger strand of the saRNA is cleaved and discarded in the cytoplasm as an active saRNA–AGO2 complex is formed. 4) The AGO2 bound saRNA is actively transported into the nucleus. 5) The active saRNA–AGO2 complex binds at the promoter region of the gene sequences (complementary DNA or noncoding RNA transcripts) and associates with the RNA helicase RHA and the RNA polymerase-associated protein CTR9. The complex subsequently associates with RNA polymerase II and activates transcription of the targeted gene. 6) The nascent RNA is produced and exported into the cytoplasm to be translated.

While nucleic acids can be remarkably effective as drugs, they show, in their native form, low serum stability and poor affinity with the cellular membrane (both are negatively charged with mismatched hydrophilicity/hydrophobicity), which highlights the relevance of introducing chemical modifications to address these shortcomings. Over the years, numerous modifications have been introduced to improve on ASO stability, efficacy, and delivery (Fig. 1(A)). The scope of modifications has been thoroughly described elsewhere (see recent reviews [10,11]). Briefly, the main modifications adopted in the field rely on (i) substituting the phosphodiester (PO) backbone unit with a phosphorothioate (PS); (ii) modifying the 2’ position of the ribose unit; or (iii) introducing an artificial biopolymer scaffold, such as phosphorodiamidate morpholino (PMO) or peptide-nucleic acid (PNA). In contrast to PO oligonucleotides, PS oligonucleotides have *Rp* and *Sp* chiral phosphorus atoms. The PS stereochemistry is not controlled in standard solid-phase synthesis, which results in the production of stochastic, 2 ^n^ diasteroisomeric mixtures, where *n* is the number of chiral phosphorus atoms in the ASO sequence. Recent synthetic developments [12,13] have facilitated the access to stereo-defined oligonucleotides and to their biological evaluation, but the emerging literature is not unanimous. In some reports stereodefined ASOs improved biological activity [14], but others are less encouraging [15]. In the gapmer ASO design (chimeric antisense oligonucleotides that contains a central block of deoxynucleotide monomers sufficiently long to induce RNase H flanked by modified bases and linkages), particular stereodefined patterns are beneficial when used with other modifications in the gap or wing region [15–17]. Several generations of constrained ribose and bridged nucleic acid modifications have also been described in the literature. They display superior binding affinity to RNA, increased nuclease resistance and improved lipophilicity in comparison to simpler 2’-*O*-alkyl modifications. Successful examples are locked nucleic acid (LNA), constrained ethyl (cEt), ethylene bridged nucleic acid (ENA), and tricyclo-DNA (tcDNA) modifications.

Importantly, incorporating these modifications can have a significant impact on the biodistribution of ASOs to tissues. For instance, systemically injected tcDNAs are reported to cross the blood–brain barrier [18]. Regardless of their class, systemically injected ASOs traffic mainly to the liver and kidney [19]. In the clinic, this translated into to the successful development of ASOs for liver targets (i.e. mipomersen, inotersen, and miravirsen). However, local delivery strategies remain necessary in the case of organs protected by biological barriers (e.g. the eye with pegaptanib or the spinal cord with nusinersen). In recent years, the Yokota group introduced heteroduplex oligonucleotides (HDOs) as a new strategy for the delivery of antisense gapmer or steric-blocking oligonucleotides [20]. HDOs consist in an antisense or antimiR sequence hybridized to a complementary RNA [20] or DNA [21] strand incorporating a lipophilic moiety. HDOs show improved biological activity *in vivo* in comparison to their single-stranded counterparts [21]. The same group recently reported in an *in vitro* study that HDOs are distinctly released from early endosomes [22]. Overall, HDOs might hold potential for delivery of ASOs to difficult-to-access cells and tissues [23,24].

Another very prevalent approach to improve the delivery of ASOs consists in attaching cell-penetrating or targeting ligands through covalent conjugation to peptidic, proteic or small molecule moieties. A first example is cell penetrating peptides (CPPs). CPPs are short, 5–30 amino acid peptides that enter cells or tissues through various mechanisms [25,26]. However, peptide-oligonucleotide conjugates (POCs) have not fully delivered on their promise yet. Advances have been made using PMO chemistry, but the development of charged PO- and PS- POCs has proven more challenging. A major synthetic issue is aggregation between the negatively charged ASO backbone and the cationic CPP, as conjugation typically is conducted with a CPP excess [27,28]. Another concern is the carry-over of undesired free molecules of CPP in the final POC [29]. Recent synthetic improvements have been reported [30,31], but it is no surprise that the most advanced peptides for delivery of PS ASOs are weakly charged (e.g. GLP [32] and neurotensin peptides). Antibodies (Abs) are another class of ligands with great potential for ASO delivery. Of particular interest are antibodies binding membrane receptors or transporters, which can be internalized and deliver a therapeutic payload within cells and tissues. The field of antibody-oligonucleotide conjugates (AOCs) is nascent but has the potential to enhance ASO delivery beyond biological barriers and to specific cellular populations. A main antigen emerging from the literature is transferrin receptor 1 (TfR1), whose AOCs show potential for muscle delivery [33]. Other antigens have been explored for cancer [34,35]. As monoclonal Abs are very large biomolecules compared to the nucleic acid therapeutics they are intended to deliver, recent research efforts are focused on conjugation to shorter Fab’ fragments [33] or to single-domain antibodies [36]. Small-molecule ASO conjugates using haptens are also under investigation. Finally, other types of ligands are being explored to improve the delivery of nucleic acid therapeutics. One example is the *N*-acetylgalactosamine (GalNAc) modification, which improves delivery to hepatocytes through asialoglycoprotein receptor mediated internalization. The GalNAc modification was initially deployed for siRNAs and is described later in this work. GalNAc ASO conjugates are investigated in several antisense pre-clinical and clinical programmes [37]. Albeit less advanced, folate is also of interest for delivery to cancer cells [38].

Ultimately, ASO efficacy relies on entering the desired cell population and trafficking to the right cellular compartment to bind its target mRNA. In most cases, following delivery and cellular uptake, an ASO is trafficked from the early endosome to the trans-Golgi network or via multivesicular bodies to the late endosome and finally to the lysosome for degradation, resulting in low levels of the ASO in the cytoplasm or nucleus where the cell’s nucleic acid targets are found [11,39]. Escape from endosomal compartments is thus critical for ASO to interact with their target gene in the cytosol or nucleus and elicit their therapeutic effect. One approach to address this consists in using small molecule enhancers (e.g. Retro-1 [40] and UNC10217938A) [41]. Alternatively, ASOs can be taken up by cells without the need for transfection reagents, a process called gymnosis [42]. Gymnosis occurs at high ASO concentrations, typically in the µM range. However, productive uptake – the efficiency of the ASO at entering the cell and modulating the expression of the target gene or protein – is very different across cell lineages. Additionally, the presence of conjugated moieties to improve tissue targeting may also impact ASO release. For example, studies on GalNAc conjugates have revealed specific cell retention mechanisms, resulting in desirable pharmacological duration effects [43].

Importantly, the bulk level of internalized ASO do not necessarily correlate with knockdown efficiency [44]. Sensitive detection methods that enable quantitative measurements of the internalized ASOs’ productive fraction are limited. Escape of siRNAs from endosomes into the cytosol has been estimated to be 1–2%. Thus, it was initially assumed that a very small fraction of internalized oligonucleotides is required to achieve an effect [45]. However, another study combining microinjection of LNA-modified ASOs into the cytosol with fluorescent imaging predicted that ~10^5^ molecules achieve over 50% reduction of a target mRNA, suggesting that a substantially larger fraction of ASO escapes into the cytosol than previously estimated [46]. A method that has the potential to clarify this further is nanoscale secondary ion mass spectrometry (NanoSIMS), which was recently shown to detect 5-bromo-2’-deoxythymidine (5-BrdT) modified ASOs with spatial resolution in cells [47]. Overall, cellular uptake and intracellular trafficking events are dictated by interactions with a range of cellular proteins that determine the efficacy of the ASO. Differences in cellular proteins might form the basis for productive or non-productive uptake. So far, about 80 proteins affecting localization and ASO efficacy have been described and recently reviewed [48–50]. Among those are cellular surface proteins like Stabilin 1 and 2 [51] and the epidermal growth factor receptor (EGFR) [52], all suggested to mediate productive uptake. Proteins that affect endosomal escape and intracellular trafficking, like the mannose-6-phosphate receptor (M6PR) [53], are also suggested to facilitate PS-modified ASO release from late endosome. So far, one of the best described ASO–protein interactions is the complex between human positive cofactor 4 (PC4) and a full PS 2′-O-methyl DNA gapmer ASO, for which a crystal structure of the DNA-binding domain of PC4 in complex with the ASO has been reported [54,55]. Further insights into protein interactions and the role of ASO sequence and 3D structure in these interactions is expected to facilitate the rational design of more efficacious ASOs.

Following the 1998 approval of fomivirsen, the first-of-its-kind PS DNA ASO, it took almost 15 years of research for the ASO field to expand. From this work, a stunning total of eight ASOs have been FDA-approved since 2013, with casimersen (Sarepta Therapeutics) being the newest as of February 2021. Despite the now firmly established success of ASO therapies, challenges related to delivery remain, and further research efforts should be undertaken to develop structural modifications that optimize their therapeutic index [15] and to understand the cellular mechanisms responsible for intracellular trafficking. It is well known that a number of tissues and biological barriers are refractory to ssON uptake, but novel, tailored peptide [32] or antibody [33] conjugates may lead to significant improvements. It remains to be seen whether novel modifications such as stereodefined PS bonds or constrained ribose chemistries will succeed in the clinic and lead to approved drugs.

3.

1. Challenges and strategies to deliver functional siRNAs to tissues

Small interfering RNAs are emerging as a new class of drugs that holds great promise for the treatment of genetically defined disorders by targeting disease-causing mRNAs for degradation. Their advantages over conventional drugs include: (i) ease of design – rationally achieved based on sequence information and straightforward screening, leading to drug candidates within short periods of time; (ii) the ability to target disease genes previously considered ‘undruggable’; and (iii) unprecedented potency and duration of effect [56]. However, clinical success is dependent on their efficient delivery to disease tissues. To date, GalNAc conjugation of siRNAs has dominated the field by efficiently delivering siRNAs to hepatocytes to treat liver diseases [57–59]. However, the use of siRNAs to treat diseases beyond the liver remains challenging and several limitations need to be addressed (Fig. 1(B)).

The success of GalNAc conjugation for targeted delivery to hepatocytes has spurred the search for novel conjugates that deliver siRNAs to other tissues and organs. Moreover, this discovery has also established conjugate-mediated delivery as the clinically dominant delivery paradigm for siRNAs after systemic administration [60–62]. To be effective, this mode of delivery requires full chemical stabilization of siRNAs. To achieve this, chemical modifications that replace all 2´-hydroxyl groups, modify terminal nucleotide linkages, and stabilize the 5´phosphate are needed to maximize the *in vivo* activity of siRNAs [63]. Unmodified and partially modified siRNAs are rapidly degraded [64,65] and cleared from the circulation by kidney filtration, leading to minimal bioavailability in tissues [66]. While numerous chemical modifications have been developed in a context of antisense oligonucleotides (ASOs) [67], the double-stranded nature of siRNAs and the need of siRNAs to interact and be loaded into RNAi machinery impose limitations on the nature of the chemical modifications that can be used. Among tolerated chemical modifications, the 2´-*O*-methyl and 2´-fluoro are the most common ones used in the clinic to substitute 2’-hydroxyl groups. Both modifications are tolerated in siRNAs and do not prevent RNA-induced silencing complex (RISC) assembly and function [68]. While 2’-*O*-Methyl modifications enhance stability, increase affinity to the targeted mRNA and reduce immunogenicity, their content and position within the siRNA can affect efficacy significantly. Incorporation of 2’-O-Methyl modifications in position 2, 6 or 14 of the antisense strand and position 11 of the sense strand decreases siRNA activity [69], likely due to the sterically demanding nature of 2’-O-Methyl which interfere with RISC affinity. Furthermore, it has been demonstrated that heavy 2’-O-Methyl modified siRNAs (80% of 2’-O-Methyl modifications in total) induce better potency and duration of effect in both mice and non-human primates compared to an alternating 2’-O-methyl/2’-fluoro patterns (50% of 2’-O-methyl modifications in total) [69]. These findings demonstrate that a fine-tuning of the chemical pattern of siRNAs is essential to optimize RISC loading and activity [69]. In addition to 2’-ribose modifications, further nuclease stability is primordial to efficiently deliver siRNA to tissues, which can be achieved by incorporating PS linkages [70]. However, PS modification can impact oligonucleotide activity and toxicity [48,71]. Moreover, siRNAs that contain PS modifications at every linkage or a high PS modification content induce minimal silencing [71,72]. Therefore, siRNAs being developed in the clinic tend to have a maximum of two PS modifications at the termini [57–59]. This simple combination of backbone and sugar modification provides additional resistance to exonucleases – the primary effectors of RNA degradation – and an order-of-magnitude increase in oligonucleotide accumulation *in vivo*.

Even if 2´-*O*-methyl, 2´-fluoro and PS modifications are sufficient to improve siRNA stability, decrease immune responses and induce robust and sustainable silencing *in vivo* after systemic administration, there are concerns about siRNA toxicity induced by off-target effects and 2´-fluoro metabolites [73]. As a result, there are constant efforts to explore novel chemical modifications that can mitigate siRNA toxicity and further enhance siRNA performance. To limit off target effects and improve siRNA activity, strand selection into RISC is a critical step, where the selection of the antisense strand needs to be favoured over the sense strand. To achieve this, modulating siRNA chemical composition can be performed to alter the strands interactions with Ago2, leading to preferential loading of the antisense strand into RISC. Alteration of the siRNA backbone chemistry can also have a significant impact on strand selection into RISC. It has been reported that the chirality of PS backbones may affect strand selection, where the S and R configurations are preferred at the 3´ and 5´ ends of the antisense strand, respectively [74]. In addition, replacing PS modifications by achiral phosphorodithioate (PS2) linkages combined with 2´-*O*-methyl modifications increases the loading of modified siRNAs into the RISC likely due to favourable hydrophobic interactions with the PAZ domain, enhancing their gene knockdown ability, which led to anti-tumour activity in this example [75]. Another interesting approach consists in blocking the 5´-end of the sense strand to be phosphorylated by incorporating specific chemical modifications (e.g. using a morpholino or inverted bases) [76]. Phosphorylation of the 5′-hydroxyl of the antisense strand is crucial for siRNA recognition by RISC [77,78]. Therefore, an effective strategy to favour loading of the antisense strand consists in blocking the phosphorylation of the sense strand. The antisense strand is thus favoured as the phosphorylation site and loaded preferentially into RISC, minimizing off-target effects. The whole sugar ring can also undergo modifications to maximize RISC loading. Unlocked nucleic acids (UNA), with higher flexibility due to unconnected 2´ and 3´ carbons, can block the entry of the sense strand and promote loading of the antisense strand into RISC by introducing chemical asymmetry into duplex siRNAs [79]. Similarly, glycol nucleic acids (GNA), another thermally destabilizing nucleotide when introduced at position 7 of the antisense strand, decreases off-target effects and mitigates hepatotoxicity [80].

As mentioned above, another contributing factor for siRNA toxicity may be the presence of metabolites of 2´-fluoro monomers [73]. To overcome this limitation and improve the pharmacological properties of siRNAs, the impact of sugar modifications at positions other than C2´ on siRNA efficacy and toxicity has been investigated. However, finding novel chemical modifications that increase siRNA stability while maintaining siRNA RISC loading and efficacy is challenging. Nonetheless, it has been reported that both C4´ and C5´ modifications are well tolerated and have remarkable enzymatic stability [81]. Furthermore, incorporation of 5´-(R)- and 5´-(S)-C-methyl-guanosine unmodified at the C2’ position provides protection against exonucleases and maintains siRNA potency. Unlike 2´-fluoro monomers [82], the 5´-C-methyl substituted guanosine monomers are not mitochondrial polymerase substrates, demonstrating that this metabolite may be safe and could enhance the pharmacological profile of siRNA [83]. Surprisingly, the incorporation of a sterically constrained bicyclic 2’-fluorinated Northern-methanocarbacyclic (2´-F-NMC) nucleotide into an siRNA at position 7 of the antisense strand and 10, 11 and 12 of the sense strand was found to induce similar potency than the parent siRNA. Moreover, the 5´-triphosphate of 2´-F-NMC is not a substrate for mitochondrial RNA and DNA polymerases, indicating that metabolites are not expected to be toxic [84]. These examples demonstrate that exploring new chemical modifications is a promising path forward to develop efficient and safe siRNAs in clinic.

Even if essential to enhance stability and potency, chemical modifications are typically not enough to deliver siRNAs to a specific tissue after systemic administration. More than 85% of unconjugated fully chemically stabilized siRNAs are cleared from the body within minutes after systemic injection [62]. Similar to ASOs, GalNAc conjugation is the clinically dominant approach for siRNA delivery to hepatocytes with high tissue-targeting specificity, long duration of action, high therapeutic index, and minimal adverse effects. As of today, three GalNAc conjugated-siRNAs developed by Alnylam have been approved (givosiran [57], lumasiran [58] and inclisiran [59]). Given the wide therapeutic index and excellent safety profile of these compounds, more GalNAc conjugated siRNAs will likely be approved to treat liver-associated disorders.

However, since the delivery of siRNAs by GalNAc conjugates is limited to hepatocytes, the design of ligands that are specific to receptors expressed in tissues beyond the liver needs to be achieved to target extrahepatic tissues. Among these ligands, antibodies have been successfully designed and conjugated to deliver siRNAs via multiples receptors including transferrin receptors (highly expressed in cardiac and skeletal muscles) [33] and cell surface antigens (expressed in multiple myeloma) [85]. Recently, Avidity Biosciences showed promising results targeting and delivering siRNA to muscles *in vivo* using antibody conjugates. They demonstrated that Abs siRNA conjugates targeting transferrin receptors allow 90% mRNA silencing in muscles after a single-dose administration in mice. Even if antibodies are promising tools to achieve receptor-targeted delivery, they are large proteins with multiple functional domains, posing a challenge for the production of antibody RNA conjugates. Alternative approaches are emerging to overcome these limitations. Conjugation of nanobodies, antibody fragments that recognize specific antigens, have been explored and enable the delivery of functional siRNAs via EGFR receptors [36]. Similarly, aptamers, which can also be viewed as chemical antibodies (along with being simpler and less expensive to manufacture) can target specific proteins and efficiently deliver siRNA to specific cell types [86]. Similar to antibodies, centyrin conjugates – small, engineered proteins derived from a human protein Tenascin C – have been designed to bind with high affinity and selectivity to numerous antigens, which makes specific tumour targeting across a broad range of tumour antigens feasible. Aro Therapeutics demonstrated that the combination of tumour-targeting centyrins with chemically stabilized siRNAs represents a versatile platform for RNAi-mediated gene silencing across multiple tumour types [87]. Lipid conjugation has also emerged as a delivery platform for siRNAs after systemic administration [60]. Diverse classes of lipids, including saturated and non-saturated fatty acids, steroids, and vitamins, with or without a phosphocholine polar head group have been recently investigated [62,88,89]. It has been demonstrated that the structure of the lipid significantly impacts siRNA clearance, lipoprotein binding, tissue distribution and efficacy [62,89,90]. Even though most of the injected lipid-siRNAs accumulate in clearance organs (liver, kidney and spleen), several lipid conjugates enable functional siRNA delivery to heart, lung, fat, muscle, and adrenal gland. For example, docosanoic acid (DCA) conjugate allows robust, safe and sustainable silencing in both skeletal and cardiac muscles after systemic administration in mice [91]. These findings represent a proof of principle that lipid engineering and conjugation is a viable strategy to improve extrahepatic delivery and efficacy of therapeutic siRNAs

The current and outstanding progress in the field of RNAi therapeutics provides an opportunity to treat diseases with unmet clinical needs. The development of therapeutic platforms capable of delivering functional siRNA to specific tissues after systemic administration will likely be achieved by defining chemical modification patterns and designing conjugates that confer predictable pharmacokinetic and pharmacodynamic properties. Major breakthroughs have been achieved to treat liver diseases when using a carefully engineered, fully chemically stabilized siRNA conjugated to GalNAc. However, despite the advances in clinical RNAi drug development, it is also clear that RNAi therapeutics could substantially expand if systemic delivery to non-liver and kidney tissues becomes viable in a clinical setting. To achieve this goal, the main challenges to overcome include simultaneously avoiding both renal and reticuloendothelial clearance, enhancing tissue retention, increasing uptake in specific cell types and improving endosomal escape [92]. Further development of advanced stabilized siRNAs and well-designed delivery ligands will likely enable the effective of delivery of siRNAs to an increasing number of extrahepatic tissues. The continued progress and development of this drug category progress will no doubt lead to many more breakthroughs in the future.

## II. Emerging RNA-based technologies

### ASOs as programmable antibiotics

Broad-spectrum antibiotics that act on a wide range of disease-causing bacteria, for example, Gram-negative or Gram-positive species, have saved millions of human lives and remain amongst the most important drugs in modern medicine. However, there is a growing need for species-specific antibiotics, due to the emergence of multi-drug resistance and the severe side effects of necessary long-term treatment with broad-spectrum antibiotics for chronic infections or in clinical settings. In addition, non-selective antibiotics harm the endogenous microbiota and can cause dysbiosis. Finally, a means to eliminate individual species in a complex community is needed to interrogate the individual contributions of the thousands of different bacteria that make up our microbiota in health and disease.

Of several promising species-specific antimicrobial candidates available, RNA-targeting antimicrobials in the form of short ASOs that inhibit essential genes on the RNA level are a promising technology (Fig. 1(C)). Such ‘programmable RNA antibiotics’ directed against mRNAs were pioneered in *E. coli* using ASOs in the 9-mer to 12-mer range that repress the synthesis of the essential fatty acid biosynthesis protein, AcpP [93]. As compiled in recent reviews [94,95], ASO-based antimicrobials have since been tested in more than a dozen diverse Gram-negative and Gram-positive bacteria, which include proof-of-concept studies in the mouse.

Most of these studies have used PMO and PNA oligomers as the antisense modality, which is then designed such that it sequesters the ribosome-binding site (RBS) of an essential mRNA of interest, preventing its recognition by the 30S ribosomal subunit and hence, protein synthesis (Fig. 1(C)). The optimal length for an antibacterial ASO seems to be in the range of 10 to 12 bases [96–98]. For example, a 10-mer anti-*acpP* PNA was found to kill bacteria most effectively in a *E. coli* model [96]. If too short, the strength of the base pairing interactions of the ASO might be insufficient to compete with ribosome binding; however, longer ASOs tend to show less efficient cellular uptake. The site of translation initiation is seen as the optimal mRNA target region for antisense repression, as evidenced from several studies testing this systematically through scanning of the 5’ regions of different mRNAs with antisense PNA or PMO [96,99]. The results from these studies demonstrate that an ASO antisense to the start codon and perhaps part of the Shine-Dalgarno sequence (SD) will be the most potent. Yet, based on observations with endogenous bacterial regulatory small RNAs (sRNAs), it may also be possible to effectively target mRNAs in other regions, for example, the 5’ UTR or the coding sequence [100], where sequence diversity is much higher. Further developments on this finding would vastly extend the target space of programmable RNA antibiotics, especially when targeting individual bacteria in complex communities.

Since ASOs poorly penetrate the bacterial envelope on their own, they are generally tethered to a short (<30 amino acids) CPP, predominantly cationic or amphiphilic in nature [101]. An 11-mer ASO is typically in the range of 3–4 kDa, whereas porins, which are the main entry gates in the outer membrane of bacteria, exclude molecules >600 Da. As a result, CPP can reduce the minimal inhibitory concentration (MIC) of a toxic ASO from millimolar to micromolar concentrations or below, thereby endowing them with the same potency as conventional antibiotics [102]. Many CPPs can penetrate both mammalian and bacterial cell membranes, which increases their attractiveness for targeting intracellular pathogens. In addition, different peptides penetrate different bacteria with different efficiencies, as illustrated by the clear differences seen between *Burkholderia* versus *Pseudomonas* and *Acinetobacter* [103–105], and some peptides seem to work better in Gram-positive than Gram-negative species (discussed in [106]), showcasing that species-specific killing in complex communities can be improved by using the most selective peptide for a target bacterium of interest.

Resistance is a concern for any antibiotic but is poorly understood with respect to antibacterial ASOs. Resistance screens in *E. coli* suggest that peptide conjugates of both PMO and PNA are taken up via the inner membrane peptide transporter, SbmA [107,108]. Yet, SbmA is necessary for only a subset of peptide-ASO conjugates and the transport mechanisms of many other constructs used in the field remain to be elucidated [108,109].

In summary, there is ample demonstration that ASO drugs can effectively eliminate diverse bacteria in complex organisms as illustrated by experiments carried out in animal models. Yet, many fundamental questions need to be addressed for this technology to reach its full potential in the clinic, but also to develop it as a tool for precision editing of the microbiome. To date, most studies of antibacterial ASOs have been end-point driven, primarily focused on determining their MIC to assess their antimicrobial activity. However, target selectivity *in vivo* and overall effects of ASO conjugates on bacterial cells have not been assessed to a significant extent. In addition, translational inhibition has been assumed to be the primary mode of action of antibacterial ASOs, but whether this is the only potent mechanism of action of these ASOs remains to be elucidated. Most recently, state-of-the-art transcriptomics by RNA-seq have been introduced and show on the global level that blocked protein synthesis selectively affects the level of the target mRNA. Such a global approach will pave the way for a comprehensive assessment of ASO specificity, off-target effects, and efficiency *in vivo*.

### Small activating RNAs

In contrast to ASOs and siRNAs, which silence gene and protein expression, RNA therapeutics can also be designed to activate gene expression. Small activating RNAs (saRNAs), which are 21-nucleotide long double stranded RNAs (dsRNAs) complementary to the promoter region of a targeted gene, are now recognized as having the capability to activate endogenous genes via an RNA-based promoter targeting mechanism (Fig. 1(D)). In 2006, Li et al. showed that dsRNAs designed exogenously to target the promoter of human e-cadherin, p21 and VEGF genes were able to elicit transcriptional activation of these genes following liposomal transfection in human cell lines [110]. The mechanism was shown to be sequence specific and dependent on Argonaute 2 (Ago2). The same observation was also confirmed in other mammalian species [111] and *in vivo* by injection of lentiviral shRNA targeting mVEGF-A promoter into mice [112]. Therefore, the ability of exogenous saRNAs to activate targeted genes in rodents and in non-human primate cells demonstrates the cross species conservation of an RNA-driven transcriptional activation that targets the promoter region of genes.

The activity of saRNAs is driven by the saRNA seed, and saRNAs need a full seed complementarity with their target to trigger upregulation [110,113,114]. However, the presence of mismatches in the antisense strand outside of the seed region can be well tolerated, where up to three consecutive mismatched nucleotides outside the seed have been shown to have little effect on saRNA activity [113,115]. In cell models, transcriptional upregulation can be observed 48–72 hours after transfection of saRNAs [110,116]. In terms of mechanism of action, saRNAs induce an increase in nascent mRNA, as determined by nuclear run-on assays, which also confirm that the observed upregulation in mRNA is not due to other effects such as increasing mRNA stability. The induced transcription activation is also long lasting, with mRNA upregulation detected as far as 12 days after transfection [114,115,117]. RNA activation has also been linked to epigenetic modifications with several reports describing the demethylation of H3K9 and di-methylation and tri-methylation of H3K4 [110,112,116]. Nevertheless, it is still unclear if the epigenetic modifications observed are part of the saRNA mechanism or a consequence of it.

While saRNAs have been shown to associate with genomic DNA at the promoter region [113,117], it is generally understood that saRNAs target promoter-associated noncoding RNA transcripts [114,118–120]. The use of controls blocking the potential cleavage induced by dsRNA duplexes demonstrate that after being loaded in Ago2 the saRNA does not induce cleavage of a potential complementary RNA target [110,114,119,120]. Instead, the saRNA-loaded Ago2 complex binds to its seed target site at the promoter region and recruits RNA polymerase 2 [113,114,116–121]. In addition, the saRNA-Ago2 bound complex has been shown to associate with RNA helicase A (RHA) and CTR9, which were both identified as crucial accessory proteins for RNA induced gene activation [117,122]. HNRNPs (heterogeneous nuclear ribonucleoproteins) were also shown to bind to the saRNA complex at the gene promoter sites. Together with Ago2, RHA, CTR9 and other co-factors, this complex is now termed the RNA-induced transcriptional activation (RITA) complex. Further characterization of the components of the RITA heteroprotein complex will undoubtedly bring to light more mechanistic features specific to saRNAs.

Since they are small oligonucleotides capable of targeting a specific gene, saRNAs represent a very attractive therapeutic tool, offering the delivery possibilities of small oligonucleotides with the unique effect of upregulating a protein of choice. In terms of therapeutic development, saRNAs have thus far been predominantly developed to re-activate tumour suppressor genes in multiple types of cancers (the reader is invited to consult a recent review by Yoon S. and Rossi J. J. which describe the advances within this field [123]). More recently, the scope of saRNA therapy has widened, with the possibility of treating genetic diseases by targeting haplo-insufficient genes. Similarly, metabolic diseases may also benefit from RNA activation. For example, targeting HNF4A with saRNAs is a promising approach for the therapeutic reversal of non-alcoholic liver disease (NAFLD) as demonstrated in an animal model [124]. Excitingly, the first saRNA therapy tested in humans is targeting the promoter for CCAAT/enhancer-binding protein alpha (CEBPA), a master regulator of differentiation of myeloid cells. In this study, the CEBPA saRNA is encapsulated in a Nov340 liposome for systemic delivery. The drug named MTL-CEBPA is now entering Phase 2 clinical trials for patient with advanced hepatocellular carcinoma (HCC) and has been shown to be safe, with no maximum dose reached in the first-dose escalation study [125,126]. MTL-CEBPA is currently assessed in patients with solid tumour in Phase 1A/B study in combination with the checkpoint inhibitor pembrolizumab [127]. saRNAs offers a very interesting tool for therapy with the possibility to upregulate gene expression with less delivery hurdle than mRNA or CRISPR activation. Further understanding of the RNA activation mechanism and pathways will give the tool to develop potent and long-lasting saRNAs, building up on shoulder of the siRNA technology.

### Nucleic acids nanotechnology

Research over the last decade has shown that the biological outcomes of nucleic acid (NA) therapeutics can be modulated by controlling their properties at the nanoscale. This is a significant advantage considering unmodified NAs are rapidly degraded in serum, can be immunogenic and typically show poor cellular uptake. By harnessing the programmable base-pairing alphabet of NAs and their fully addressable synthesis, NA nanostructures can be designed with control over the geometry, size, and positioning of ligands. These constructs can readily enter cells, carry various cargoes, and perform functions outside and inside cells. They have been extensively studied and validated as gene regulations agents, detection agents, immune regulation agents, and cancer vaccines, both *in vivo* and *in vitro*. We will discuss the advantages, challenges, and opportunities available to NA nanostructures for biomedical applications.

**Figure 2.**
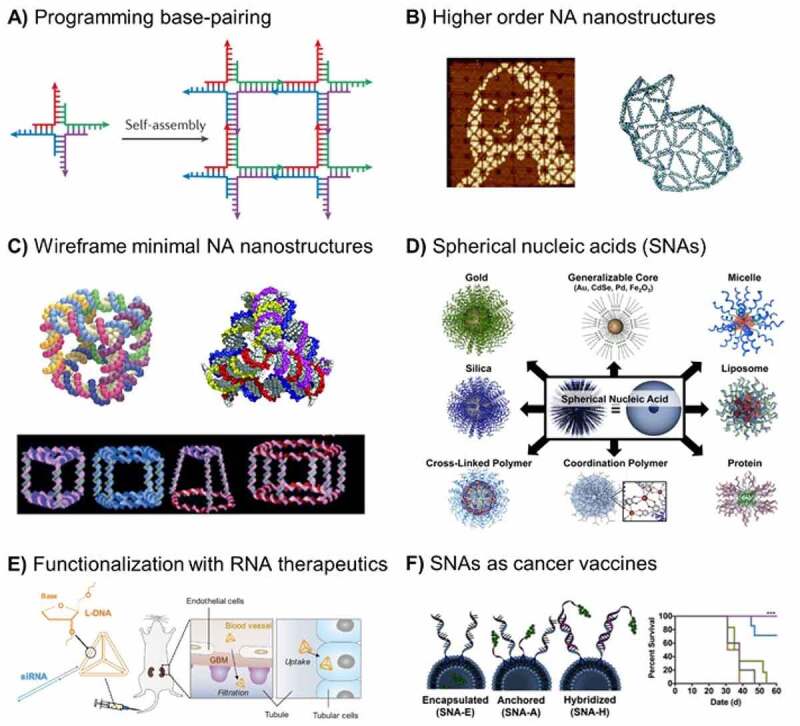
Nucleic acid nanotechnology. (A) Programmable assembly of DNA strands into a 4-way junction with single-stranded toehold regions that promote assembly into higher order structures [128]. (B) Assembly of higher order structures using the programmable base pairing alphabet of DNA. On the left, representation of the Mona Lisa using fractal assemblies of DNA origami [130]. On the right, 3D DNA rabbit assembled from DNA polyhedral meshes [131]. (C) Wireframe minimal 3D NA acid nanostructures in the shape of a cube, a tetrahedron and various prismatic structures [128,141]. (D) Spherical nucleic acids with and amenable cores for nucleic acid functionalization [145]. (E) Functionalization of a L-DNA tetrahedron with siRNAs. This construct was found to accumulate in the kidneys and mediate gene knockdown in vivo [149,150]. (F) SNAs as cancer vaccines. The nanoscale organization of the antigen (green) and adjuvant (blue) determines the efficacy of the overall construct [147].

The power of NA nanotechnology resides in the complete control of the user over the structure and function of the resulting structures [128]. NA self-assembly is a predominant approach to generate complex structures that relies on base pairing interactions to form well-defined structures. This is achieved through the precise programming of oligonucleotide sequences and their interactions with complementary partners through hybridization (Fig. 2(A)). Single-stranded toehold regions are another important design element since they can be used to synthesize expanded networks, 3D nanostructures or responsive systems. Typically, NA constructs can be prepared in quantitative yields, by mixing all strands in cation-containing buffers, followed by a thermal anneal to promote the self-assembly of a monodisperse product. NA nanostructures are typically constituted from 3 to 1000s of strands, enabling the design of complex anisotropic structures with responsive parts. A wide variety of nanostructures, ranging from a few nanometres to microns in size, with simple polyhedral shapes, to more sophisticated 3D structures can be routinely achieved [128,129]. These include complex structures such as a reproduction of the Mona Lisa (fractal assembly) or the assembly of a 2D dolphin (DNA origami) or even a 3D rabbit (polyhedral meshes) (Fig. 2(B)) [130–133]. These examples illustrate how NA technology has pushed the limits of complexity at the nanoscale by enabling the design of almost any structure at low-cost and using simple algorithms in highly automated fashion [134]. Wireframe NA minimal nanostructures have also been assembled in an effort to retain 3D shapes while reducing the complexity and number of strands required (Fig. 2(C)) [135,136]. The lowering costs of oligonucleotides, combined with the robust methods and free software now available for the design of NA-based nanostructures make this technology readily accessible to a wider scientific community [137]. Recent efforts to expand the scope of NA nanostructures have led to their functionalization with synthetic inserts that promote orthogonal non-covalent interactions to expand their self-assembly properties [138]. Overall, NA nanostructures can be designed with various shapes and functionalities and offers a platform to build clinically relevant delivery systems [139–141].

Spherical nucleic acids are another prevalent architecture in NA-based nanomaterials [142]. They consist of a dense shell of radially oriented oligonucleotides covalently functionalized onto a nanoparticle core (Fig. 2(D)). In this nanoscale architecture, SNAs gain novel properties that are absent in the oligonucleotides from which they are derived. For example, SNAs have been demonstrated to rapidly enter cells by engaging scavenger type A receptors, in addition to showing reduced immunogenicity and improved serum stability [143]. Synthetically, the power of this approach resides in the requirement for few unique sequences, which get functionalized onto nanoparticle cores using commercially available materials and scalable protocols accessible to non-experts [144]. In contrast to self-assembled NA structures, spherical nucleic acids are isotropically functionalized with approximately 100 copies of one or two unique sequences onto their nanoparticle core, conferring them inherent multivalency but fewer unique sites to modify. This facilitates their synthesis, which can be readily scaled up for *in vivo* experiments. As a result, SNAs have been developed into a very versatile platform and include many types of cores (e.g. gold nanoparticles, proteins, liposomes) (Fig. 2(D)) benefitting from the large array of reactive groups available to functionalize ends of oligonucleotides (e.g. thiols, amines, strained alkynes, and cholesterol) [145]. Spherical nucleic acids are currently being translated to the clinic for a wide range of diseases with unmet therapeutic needs [145].

One of the main advantages of the use of NA structures is the unprecedented control on the nanostructure size and shape, and the positioning of ligands. Structures can be functionalized with nucleic acid drugs and biologically relevant targeting moieties via post-synthetic modifications and non-covalent interactions at precise location [137,146]. Through these approaches, aptamers, proteins, small molecules, antibodies, and peptides have been found to modulate the properties, of NA structures, for example, by improving their targeting ability and potency [137,147,148]. Importantly, RNA therapeutics can also be readily attached using covalent linkages or hybridization strategies, while retaining their potency *in vitro* and *in vivo* (Fig. 2(E)) [149–151]. Interestingly, their natural compatibility with NA nanostructures has facilitated the development of monodisperse delivery systems entirely NA-based. As an example, DNA nanoparticles have been successfully attached with siRNAs and tumour growth inhibition was observed at doses where the siRNA alone was not effective [152].

Moreover, NA technology enables the assembly of multivalent constructs with increased relevance for biomedical applications, a challenging feature for other nanoparticle systems. In the case of SNAs, their dense multivalent oligonucleotide shell of promotes the rapid cellular uptake of their nanoparticle core [143]. In a recent example, this strategy was used to promote the delivery of a functional enzyme to tissues in a mouse model [153]. Very importantly, the precise control over the nanoscale architecture can give rise to more potent NA-based therapeutics. In a recent example, the precise control over antigen and adjuvant placement on an SNA was found to significantly enhance the potency of cancer vaccines *in vivo* (Fig. 2(F)) [147]. In NA-based nanostructures, the programming of unique-binding sites enables the selective attachment of ligands in controlled patterns, making it possible, for example, to design and assemble complex anisotropic patterns that are difficult to achieve using other strategies [154]. In a recent example, two aptamers were attached on a DNA nanostructure to effectively prevent thrombosis *in vivo* [155]. Other self-assembled ‘smart’ delivery systems have also been used to deliver multiple drugs (dual therapy) or to exploit the synergistic effects of different ligands. For example, oligonucleotide and small molecule therapeutics (e.g. doxorubicin) have been combined for increased *in vivo* efficacy in a cancer model [156]. Finally, both SNAs and NA structures can be designed with dynamic features, to conditionally release their payload, upon recognition of sequences in physiological environments, increasing their relevance for biomedical applications [157–159].

While it is known that shape and size can influence tissue distribution, rules for precise organ targeting of nanomaterials remain difficult to extrapolate. NA nanostructures can help address this challenge since they are easily designed and thus provide an ideal and versatile platform to understand 3D structure-activity relationships of nanomaterials. In a recent example, the effect of four different shapes, and four different backbone chemistries on the biodistribution of constructs *in vivo* was studied to determine the ideal cancer drug carriers [160]. Additionally, since NA-based structures can easily be tuned in terms of ligand valency, positioning, and density, they have enabled systematic studies to determine which synthetic factors contribute most to a desired biological property, greatly enhancing rational design capabilities [161]. While linear NA delivery has remained mostly limited to the liver, 3D NA structures might offer new alternatives for delivery, as their distribution profiles differ from linear oligonucleotides, which is expected to facilitate applications targeted to the kidneys, tumour, skin, etc. [137,145]. Besides answering questions regarding nanotechnology, NA constructs can also answer fundamental biological questions, such as receptors organization [162], cell interactions [163] or intracellular processes [164,165]. For example, it was recently shown that compaction and cholesterol tagging of structures could increase specificity towards certain blood cells [166], or that mechanical properties could promote tumour accumulation [167].

It has now become possible to routinely produce NA constructs at scale for *in vivo* experiments. However, more research efforts need to focus on the characterization of these constructs in physiological conditions to propel them towards clinical applications. The ongoing development of experimental methods to understand the fate of NA structures in terms of biodistribution, protein interactions, cellular uptake, and mechanisms of action represents a very promising strategy to achieve these goals and will facilitate the design of the next generation of constructs [168]. One outstanding challenge towards designing effective NA-based constructs for biological applications is the requirement for structures with improved resistance to enzymatic degradation [169]. While assembling strands into 3D constructs improves nuclease resistance compared to linear oligonucleotides, many NA-based constructs still suffer from low circulation times and thus a reduced ability to reach their target site. Towards this, it has been shown that NA nanostructures benefit from the development of chemical modifications in the field of nucleic acid therapeutics (2ʹF, LNA, etc.). For example, introducing L-DNA, the mirror image of natural DNA, has greatly improved the stability of DNA structures in physiological conditions going from a few hours to several days [170]. Interestingly, since nucleic acid constructs act as carriers for other therapeutics, they do not need to be bioactive and thus are good substrates for chemical modifications that might otherwise alter their therapeutic effect. As a result, chemical modifications that improve stability, biological tolerance, and pharmacodynamics, can all be introduced without having to account for retained therapeutic activity. This opens a wider chemical space to explore to design more effective nucleic acid-based drug carriers.

The escape of NA constructs from endosomal compartments upon cellular uptake, is another very important challenge for the field. After cellular uptake, most NA nanoparticles remain trapped in endosomes where they typically degrade over time, preventing them from reaching important intracellular targets such as mRNA and genomic DNA. This limits their potency in therapeutic applications. However, recent reports suggest that the 3D structure of NA-based constructs, the use of chemical modifications and nanostructure design can play a big role in promoting endosomal escape. For example, NA-based structures have been shown to interact with lipid bilayers to drive their successful uptake and endosomal escape [171,172]. Small molecules modifications or attachments have also been used to promote endosomal escape [149]. The development of generalizable strategies to promote endosomal escape will be critical to establish nucleic acid-based nanostructures as a versatile platform for drug delivery.

Finally, more insights into the *in vivo* behaviour on NA-based constructs are needed for them to reach clinical maturity. Understanding the role of their surface charge, size, shape, aspect ratio, and placement of ligands will be critical in developing constructs with desired *in vivo* characteristics. Among these, carefully analysing the immunogenic profile of NA structures will be an important step in developing safe carriers and to better harness their properties to generate more effective therapeutics [173,174]. Additionally, the protein corona, the protein layer formed upon injection into biological fluids, will also need to be carefully assessed, as it can play a major role in dictating the fate of particles [168]. The development of methodologies dedicated to nucleic acid-based therapeutics will be crucial to achieve these goals. This will be important since the nucleic acids nanotechnology field offers unprecedented opportunities to answer fundamental questions related to nucleic acids stability, delivery, and potency [175]. Taken together, the recent achievements in nucleic acid-based nanostructures and SNA development showcase their high level of promise as versatile drug delivery vehicles for NA therapeutics.

### Viral prohead RNA

**Figure 3.**
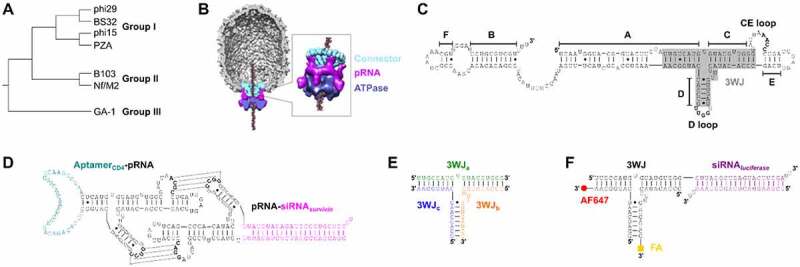
(A) Phylogenetic tree depicting the branching of the phi29-like phage genus into groups I, II, and III. Adapted from [176]. (B) Cryo-electron microscopy reconstruction of the phi29 phage packaging motor. The prohead is grey. The multimeric motor components are labelled: connector (cyan), pRNA (magenta), and ATPase (purple). Adapted with permission from [178]. (C) Primary and secondary structure of the phi29 pRNA. Helices and kissing loops are labelled. The three-way junction (3WJ) is highlighted in grey. Lines are Watson-Crick base pairs. Dots are wobble pairs. Adapted from [181]. (D) Dimer of pRNA chimeras. A CD4-binding aptamer is teal, a survivin-targeting siRNA is magenta, and pRNA scaffolds are black. Intermolecular kissing loop interactions are indicated with dashed lines. Adapted from [190]. (E) The pRNA 3WJ assembles from three component strands: 3WJ_a_ (green), 3WJ_b_ (Orange), and 3WJ_c_ (blue). Adapted from [194]. (F) A 3WJ (black) is functionalized with a luciferase-targeting siRNA (purple), folic acid (FA; yellow square), and Alexa Fluor 647 (AF647; red circle). Adapted from [196].

Prohead RNA (also known as packaging RNA; pRNA) is a noncoding RNA produced by phi29-like bacteriophages (phages) (Fig. 3(A)) [176]. During phi29-like phage replication in host bacteria, pRNA assembles with itself, connector proteins, and ATPase proteins to form a molecular motor that packages viral genomic DNA into immature capsids, or proheads (Fig. 3(B)) [177,178]. The pRNA is required for DNA packaging [179], but its function remains unknown. It is speculated that in other phage packaging motors, a protein has assumed the role of pRNA, consistent with the later stages of the RNA world hypothesis and a transition from an RNA world to an RNA-protein world [180].

Phylogenetically related pRNAs share a common secondary structure consisting of six helices, several bulge loops, two kissing loops, and a three-way junction (3WJ) (Fig. 3(C)) [181]. Intermolecular base pairing interactions between the kissing loops mediate pRNA self-assembly on the viral prohead [182]. Interestingly, pRNA also self-assembles *in vitro*, with some sequences capable of forming dimers, trimers, and higher order multimers [183,184]. The 3WJ is an important contributor to pRNA self-assembly. Indeed, pRNA 3WJs have a wide range of thermodynamic stabilities [185]; exchanging an unstable 3WJ for a stable 3WJ can direct pRNA to self-assemble into larger multimers *in vitro* [184,186].

Several proof-of-concept studies have harnessed pRNA self-assembly to achieve targeted delivery of siRNAs [187–189]. In one pioneering study, Guo *et al*. created chimeras by modifying the phi29 pRNA with a CD4-binding aptamer, an siRNA targeting *survivin*, or folic acid (FA) [190]. The authors then paired the chimeras *via* the mechanism of pRNA dimerization. siRNAs were released from pRNA-siRNA chimeras by the ribonuclease Dicer *in vitro* [190]. An aptamer-pRNA:pRNA-siRNA dimer (Fig. 3(D)) induced cell death in CD4-overexpressing cells, whereas the individual monomers were inert [190]. Moreover, pre-treatment of folate receptor-positive KB cells with an FA-pRNA:pRNA-siRNA dimer suppressed tumour formation in athymic nude mice receiving KB tumour xenografts [190]. This co-delivery approach has since been extended to different pRNA stoichiometries [191], viral pathogens [192], and oncogenic targets [193].

Other proof-of-concept studies have leveraged the pRNA 3WJ, specifically, for targeted siRNA delivery. In a seminal publication, Shu *et al*. assembled the pRNA 3WJ from three component strands *in vitro* (Fig. 3(E)) [194]. The 3WJ formed in the absence of metal salts, remained associated at low concentrations, and exhibited a high thermodynamic stability [194]. *In vivo*, the blood half-life measured for a 2’-F 3WJ was over 6.5 hours, compared with less than 5 minutes for a 2’-F siRNA [194]. Furthermore, Abdelmawla *et al*. have reported that the 3WJ is non-toxic and non-immunogenic, with favourable biodistribution and pharmacokinetic profiles in mice [195]. Notably, systemically administered 2’-F FA-3WJ-siRNA molecules (Fig. 3(F)) accumulate and are active in normally inaccessible brain tumour tissue [196]. Promising results have also been reported for FA- or aptamer-3WJ-siRNA molecules in human gastric [197], breast [198], and colon cancer xenograft mouse models [199].

The value of using the phi29 pRNA or its thermodynamically stable 3WJ as vectors for siRNA delivery lies in the platforms’ reported stability in the biological milieu and ability to integrate several functionalities in a single RNA structure. However, additional studies will be needed to help resolve the potential of pRNA as a clinically relevant siRNA delivery strategy. Specifically, it will be important for future studies to establish the pharmacokinetic/pharmacodynamic superiority of FA- or aptamer-3WJ-siRNA molecules over similar constructs lacking the 3WJ connector. Additionally, present engineering efforts are focused on the phi29 3WJ; phylogenetically related 3WJs with higher thermodynamic stabilities [185] may be better vectors for siRNA delivery. Moreover, in contrast to other synthetic RNAi triggers [200], it remains unclear how exactly chimeric 3WJ-siRNA molecules interface with the RNAi machinery to trigger gene silencing. A more detailed understanding of how these unique molecules function may help unlock their therapeutic potential.

### Oligonucleotide based artificial Ribonucleases

Sequence-selective cleavage of RNA is important to implement therapeutic strategies that aim to reduce harmful RNA levels as well as to develop tools (e.g. restriction enzymes) that can be utilized in molecular biology. As therapeutics, gapmer ASOs and siRNAs achieve this goal by taking advantage of endogenous enzymes, RNase H and RISC, respectively. The chemical architecture of these nucleic acids therapeutics is largely dictated by the need to support enzyme action, placing considerable limitations on the chemical modifications that can be incorporated. However, if greater freedom in the design of therapeutic oligonucleotides was possible, it could potentially address some of their current challenges including addressing off-target effects and sequence and chemistry-dependent toxicity [73,201]. Moreover, another potential issue that could be addressed is the saturation of endogenous RNA processing pathways from the competition between endogenous and therapeutic oligonucleotides, which can lead to non-specific toxicity [202].

**Figure 4.**
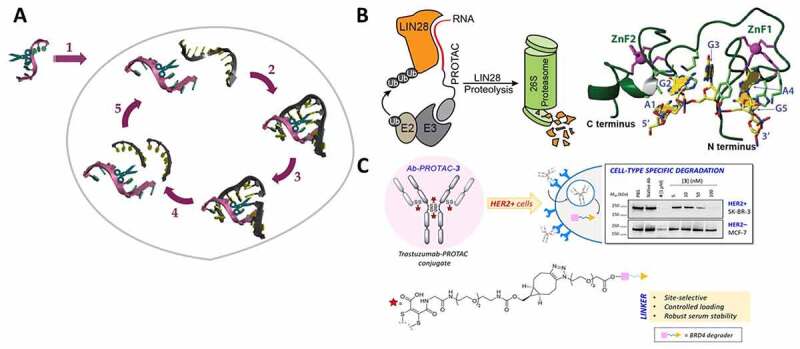
(A) Schematic presentation of the foreseen action of oligonucleotide-based artificial ribonucleases in a therapeutic setting: 1) Cell entry 2) Sequence-dependent hybridization to the RNA target by Watson-Crick base pairing 3) Cleavage of one or several phosphodiester linkages in the RNA target 4) Release of cleaved RNA fragments 5) Finding the next target to repeat the cycle and give substrate turnover. (B) Mechanism of action of RNA‐PROTACs binding RBPs and directing them to degradation. Structure of Lin28 zinc finger domain binding to its consensus sequence AGGAGAU, which was used as ligand for the PROTAC. (Adapted from Ghidini et al [228].) (C) Proposed mode of action of an antibody−PROTAC conjugate, resulting in HER2-dependent protein degradation and its overall structure. (Adapted from Maneiro et al [231].).

As a result, there has been significant interest in the development of molecular constructs capable of catalytic sequence selective RNA degradation in the absence of endogenous enzymes. These can be purely synthetic constructs, which will be covered here, or in-vitro selected ribozyme mimics, such as DNAzymes and XNAzymes (*i.e*. FANAzymes) [203], which is an alternative avenue not covered here. Synthetic constructs that can act as artificial ribonucleases contain a cleaver – also called ‘molecular scissors’ – in their oligonucleotide structure. These oligonucleotide-based artificial nucleases can recognize RNA sequences through Watson–Crick base pairing and then catalyse the cleavage of the target RNA, without requiring endogenous enzyme action [204–206]. In this process, the oligonucleotide will find the target RNA through base-pairing, form a complex with a productive structure, cleave the bound RNA with the attached ‘molecular scissors’ and then be released to find another copy of the RNA target and repeat the process (Fig. 4(A)). Numerous approaches to artificial nucleases have been developed over the years, including systems based on catalytically active metal ions (*e.g*. metal chelating groups of trivalent lanthanide ions Tm^3+^, Yb^3+^ and Lu^3+^ or divalent transition metals Zn^2+^ and Cu^2+^ among others, and metal-free systems based on moieties such as oligoamines or imidazole that can act as acid–base catalysts at physiological pH [204–206].

An important aspect for the design of artificial nucleases is that single stranded RNA is more vulnerable to cleavage than double stranded RNA [207]. The predisposition to cleavage in single-stranded RNA is likely due to the fact that conformational equilibria of ribose moieties does not pose a substantial barrier for adopting a structure that is productive for cleavage (e.g. a nucleotide in a single strand can readily adopt a conformation favourable for a 2’-hydroxyl attack on the adjacent phosphodiester in RNA). It has been suggested that an ‘in-line’ conformation is needed for this to occur [208]. In a double stranded structure, a substantial conformational change would be required to promote intramolecular attack of the 2’-hydroxyl on the vicinal phosphodiester. That would in turn lead to breaking of hydrogen bonding, thus resulting in a concomitant energetic penalty. As a result, it is beneficial to direct cleavage by artificial nucleases to single-stranded regions in RNA. Most early approaches placed the cleaver at the terminus of the oligonucleotide. However, turnover was not efficient since the base-pairing interactions were identical before and after the cleavage and thus the release of the target RNA was found to be slow. In addition, most early approaches used excess of the artificial ‘nuclease’ and preventing a quantification of turnover rates [204–206].

A more advanced concept consists in forcing the formation of a single-stranded bulge in the central region of the RNA by partial complementarity (i.e. the oligonucleotide based artificial nuclease is complementary to the RNA target on either side of the bulged out region [209]). The formed bulge will thus be more predisposed to cleavage and the ‘molecular scissors’ will then catalyse the cleavage of one or several phosphodiester linkages in the bulged-out region of the RNA target. Ideally, this should occur selectively in the desired region and only when bound to the specific target (*e.g*. not in any other nearby single stranded RNA species). After cleavage, the fragments are released (i.e. fragment binding to the artificial ribonuclease must be weaker than that of the intact RNA). This can be readily achieved if the RNA scission occurs at a central part of the RNA/artificial ribonuclease complex, leaving the cleaved complex significantly destabilized and prone to dissociation [210]. The artificial ribonuclease will then be able to find the next target to repeat the cycle and achieve turnover of the substrate. Achieving high enough cleavage rates has been shown to be a major challenge in the development of artificial ribonucleases. Moreover, depending on their design, attaining turnover with artificial ribonucleases can also be difficult.

The development of metal-dependent as well as metal-free artificial ribonucleases has a significant history which has been covered in detail in previous reviews [204–206]. Metal-free artificial ribonucleases, which do not require the presence of metal cofactors, are attractive due to their promise of greater biocompatibility. Tris(2’-aminobenzimidazole) have been utilized as metal-free ‘molecular scissors’ in several constructs based on a DNA, PNA or DNA/LNA mixmer backbones [211–213], and turnover of the substrate has been realized. [211]The observed half-lives of RNA cleavage have typically been quite limited (10–20 h) [211–213] although a 3.5-h half-life was recently reported for the cleavage of a proto-oncogenic serine/threonine kinase PIM1 mRNA fragment [212,213]. Leucine, arginine, and glycine-rich peptides have also served as ‘molecular scissors’ in oligonucleotide constructs. POCs have been designed to target the 3’ acceptor stem and TΨC arm of tRNA^Phe^, taking advantage of the bulge formation design to promote turnover but display complex cleavage patterns where, in addition to the expected cleavage sites, even distant RNA regions can be accessible for cleavage [214]. Such POCs have been used to target miRNAs, using a terminally located peptide cleaver [215] or ‘dual’ conjugates where the peptide is linking two separate oligonucleotides [216]. However, these conjugates are not independent of endogenous enzymes, since, to obtain turnover, recruitment of RNase H in needed and achieved by incorporating deoxyribonucleotide recognition motifs [215,216].

The most efficient oligonucleotide-based artificial ribonucleases reported to date are based on PNAs conjugated to metal ion chelates of Cu(II) or Zn(II). RNA targets forming 4-nucleotide bulges are cleaved at a single site with 20–30 min half-lives by Cu(II)-dependent PNA-neocuproine conjugates (PNAzymes) [217,218]. These PNAzymes are essentially artificial RNA restriction enzymes (i.e. they give turnover, high sequence specificity and even display selectivity in the bulge region of the target RNA). They exhibit excellent mismatch discrimination, which makes them less likely to cause adverse effects by acting on off-targets. In addition to the stability of PNA, a major advantage with these constructs is that further conjugation of potentially performance-enhancing entities (*e.g*. peptides) to the PNA backbone is straightforward and shown not to interfere with their activity [219]. Moreover, these PNA-neocuproine conjugates are also relatively effective in the presence of Zn(II) [220]. Zn(II)-neocuproine ‘molecular scissors’ have been used in 2’-OMe RNA-based artificial ribonucleases [209]. With both 2’-O-Me RNA and PNA backbones, the cleavage of the target RNA by the Zn(II) systems is, so far, reported to be less specific and less efficient (7–8 hour half-lives) than with their Cu(II) counterparts [209,217–220]. It is desirable to obtain at least as efficient cleavage with Zn(II) systems as with the Cu(II) systems in order to obtain better biocompatibility.

There are still several additional limitations to overcome with this technology for it to be a proper alternative to ON therapies that depend on endogenous enzymes. Delivery to cells and specific tissues is, as for all ON therapies, a major limitation. Apart from this, there are also challenges specific to oligonucleotide-based artificial nucleases that need to be overcome. Metal-ion based “molecular scissors are in general more powerful than non-metal ion-based ones, but even the former should preferably reach higher efficiency, especially for the more biocompatible Zn(II) nucleases. Metal ion-dependent systems also need a sufficient local concentration of the metal ion in the cellular environment to function. This can potentially be achieved if diseases causing significantly raised levels of metal ions are targeted. Alternatively, the use of ‘molecular scissors’ based on chelating moieties that bind the metal tightly enough to give a stable complex may be needed, but the metal ion must retain its ability to catalyse RNA scission. The relative biocompatibility of zinc makes this metal especially attractive for therapeutic artificial ribonucleases, as it can be present in micromolar concentrations and thus potentially utilized by artificial ribonucleases with a zinc binding constant in the nanomolar range [209,217–220]. Research is in progress to overcome these remaining obstacles. The strength of this technology is that it can perform sequence and site-specific cleavage of RNA. It may also be developed as an oligonucleotide therapy in a not-too-distant future. If sufficiently efficient artificial nucleases are developed, then in principle all targets that are considered for antisense gapmers or siRNA can be targets for oligonucleotide-based artificial nucleases. Of particular relevance for therapy are diseases where an accumulation of zinc ions occurs in cells as a consequence of the disease (e.g. as in malaria when the plasmodium parasite infects red blood cells). Other interesting targets are RNA viruses and miRNAs (especially those with long half-lives), which if targeted with artificial nucleases could enable both down and upregulation of mRNA.

### Nucleic acid-based PROTACs: oligonucleotides that hijack the ubiquitin-proteasome system to degrade nucleic acid-binding proteins

Under physiological conditions, a complex network that includes folding enzymes, chaperones and ATP motors, controls the elimination of misfolded proteins [221]. The two main intracellular recycling mechanisms are autophagy and the ubiquitin-proteasome system (UPS). More specifically, the UPS is responsible for the degradation of short-lived proteins and soluble misfolded proteins [222]. Proteins are marked for destruction through the covalent attachment of the small protein ubiquitin onto primarily lysine residues of the target protein. The first step of the reaction is mediated by an E1 ubiquitin-activating enzyme that activates the ubiquitin monomer. Subsequently, the activated ubiquitin is passed onto an E2 ubiquitin-conjugating enzyme, before ultimately being attached to the target protein through the action of an E3 ubiquitin ligase.

Targeted protein degradation is an emerging protein silencing strategy, which uses small molecules ligands to knock down a protein of interest (POI) by hijacking the endogenous UPS system. PROTACs are bifunctional molecules composed of a ligand that binds the POI and a ligand that recruits the E3 ligase. Upon formation of a ternary complex target:PROTAC:E3, the POI is marked with ubiquitin for degradation by the proteasome [223]. PROTACs achieve high efficiencies owing to their catalytic mechanism and by creating the conditions for energetically favourable protein:protein interactions by bringing the target protein and E3 ligase into close proximity [224]. Initial studies have focused on the degradation of hormone receptors, specifically androgen receptors and oestrogen receptors, and have recently reached clinical trial phase [225]. A reportedly orally bioavailable selective agent for the degradation of androgen receptor (SARD) with an undisclosed structure, ARV110, developed by Arvinas, was initiated in phase II trials in 2021 in a late-stage mCRPC (metastatic castration-resistant prostate cancer) patient population. Although several protein silencing methodologies, such as the ones described in the previous chapters, have been developed to regulate protein production at the RNA level, most silencing systems still suffer from limitations, including irreversible silencing protein expression through genetic ablation and misinterpretations arising from potential genetic compensation and/or spontaneous mutations [226]. Novel modalities such as Proteolysis Targeting Chimera (PROTAC) have the potential to alter the abundance of targeted proteins overcoming the hurdles of silencing mechanisms [227].

To evaluate the potential of nucleic acids based-PROTACs, it is important to contrast the advantages and disadvantages of PROTACs compared to RNA-based therapeutics. RNAi and antisense can target essential gene products and have the advantage of being active on gene families based on sequence similarities. Since their action is at the RNA level, their effect is not influenced by the protein isoform. As previously described, both technologies suffer from potential off-target effects and rely on protein turnover, thus requiring prolonged treatments. However, PROTAC is independent of regulation on DNA/RNA and can target essential proteins directly. Unfortunately, one of the major drawbacks of PROTACs is that developing a new PROTAC system is time consuming due to unpredictable structure/efficacy relationships. Although the field has recently attracted a high degree of attention, there is still a limited availability of POI tested targets and a limited number of verified E3 ligases. Proteins with catalytic activity can be successfully drugged, but most other families, such as transcription factors or RNA-binding proteins (RBPs), lack binding sites for small ligands and therefore remain challenging targets for all small molecule-based approaches, including PROTACs. As a result, it is becoming more evident that merging two very different fields, the RNAi/antisense and PROTAC to generate a new targeting tool, could extend the therapeutic capabilities of both approaches and overcome their disadvantages.

The first reported single strand oligonucleotide-based PROTAC, termed RNA-PROTAC, was introduced as a proof-of-concept for the degradation of two RBPs, LIN28 and RBFOX1 [228] (Fig. 4(B)). These new chimeric structures include a small, structurally modified oligoribonucleotide (iso-sequential to the native RNA-binding element of the RBP), which serves the function of docking to the protein RNA-binding site, and an E3-recruiting peptide, derived from the HIF-1a protein, which labels the RBP for proteasomal degradation. The Crews lab reported an oligo-PROTAC-based strategy for targeted transcription-factor degradation, TRAnscription Factor Targeting Chimeras (TRAFTACs) [229]. TRAFTACs consist of a chimeric oligonucleotide that simultaneously binds to the transcription factor of interest (TOI) and to HaloTag fused dCas9 protein. The TRAFTACs concept was tested on two oncogenic transcription factors, NF-κB and brachyury. This approach uses the artificially engineered dCas9-HT7 fusion protein as a mediator, which limits its potential use in clinic. Huang et al. reported a simpler construct, O’PROTACs, that instead docks to the target transcription factor through a double-stranded oligonucleotide [230]. O’PROTACs were tested on ERG and LEF1, two highly cancer-related transcription factors, and selectively promoted the degradation of these proteins, thereby inhibiting their transcriptional activity in cancer cells.

The combination of two very promising therapeutic modalities is now opening the possibility of targeting proteins, which were not considered druggable in the small molecule or RNA-targeted drugs contexts. As discussed in previous chapters, one of the barriers to using oligonucleotides as drugs is their inefficient delivery *in vivo*, and this weakness is not resolved by incorporation into a PROTAC. Combining nucleic acid-based PROTACs with cell type-selective delivery moieties would be a further step in nucleic acid-based PROTAC design. An interesting approach in this vein was proposed by Tate et al., who described the first antibody-PROTAC (Fig. 4(C)). The authors reported the design and synthesis of a trastuzumab-PROTAC conjugate (Ab-PROTAC 3), which degraded its BRD4 target selectively in HER2-positive breast cancer cell lines, while sparing HER2-negative cells. The E3 ligase-directed degrader activity was caged with an antibody linker, which can be hydrolysed following internalization, releasing the active PROTAC and inducing catalytic protein degradation [231]. Another innovative concept recently reported is the light-inducible switch PROTAC. Opto-PROTAC [232] enabled the degradation of protein targets in a spatiotemporal manner, by adding a photolabile caging group on pomalidomide whereas PHOTACs (PHOtochemically TArgeting Chimeras) [233] incorporate azobenzene photoswitches into PROTACs. Critically, Opto-PROTACs display no activity in the dark, and degradation can be induced at a specific time, location, and rate by ultraviolet A irradiation.

Several other protein-degradation platforms (dTAGs3 autophagy targeting and SNIPERs, Trim-Away, chaperone-mediated autophagy) have been developed in parallel to PROTACs. However, all these methods involve the manipulation of intracellular protein degradation machinery, which limit their targets to proteins that contain cytosolic domains to which ligands can bind. Bertozzi et al. developed LYsosome TArgeting Chimeras (LYTACs) [234], which broaden the target spectrum to extracellular and membrane-associated proteins, which represent 40% of all protein-encoding genes. LYTACs are made of an oligoglycopeptide moiety that binds to a transmembrane receptor (the cation-independent mannose-6-phosphate receptor; CI-M6PR) at the cell surface linked to an antibody (or small molecule) that binds to the protein targeted for destruction. The formation of the complex between the cation-independent mannose-6-phosphate receptor (CI-M6PR), LYTAC and the POI at the plasma membrane directs the complex for destruction by protease enzymes. The receptor–ligand interaction triggers the internalization of the extracellular proteins through receptor-mediated endocytosis, inducing target degradation in membrane-enclosed organelles, the lysosomes. A natural next step would be the employment of such a technology in combination with ssONs, which normally carry ligands for membrane receptors. The disadvantage of PROTAC of being unpredictable in terms of structure/efficiency due to its modularity, provides a potential opportunity for the development of a new class of nucleic acids-based therapeutics designed to be amenable for any class of POI and physiological context.

## Conclusive remarks

Oligonucleotide-based technologies have been intensively growing and have taken their place as a major therapeutic platform to address unmet medical needs. It took nearly 40 years of oligonucleotide therapeutic development for them to reach clinical utility. The 2016 approval of nusinersen and eterplirsen marked an important milestone for ASO technology, quickly followed by the 2018 approval of the siRNA patisiran. Currently, 14 RNA therapeutics are approved, and many others are in clinical development, demonstrating that this technology continues to advance at a rapid pace. However, as of today, a full understanding of antisense technology is not achieved yet. Significant adverse events such as thrombocytopenia [235] may occur in patients and thus more investigations are required to fully defined the impact of antisense technology.

Over the last two years, already three GalNAc-conjugated siRNA drugs (givosiran, lumasiran and inclisiran) have been approved and it is likely that more will be commercialized in the near future. However, terminations of nucleic acid-based therapeutics clinical trials (e.g. revusiran) demonstrate that a better understanding of the impact of chemical modifications on activity and toxicity needs to be addressed to further develop robust drugs in clinic. Furthermore, while GalNAc drugs enable effective targeting of the liver, efficient delivery to tissues beyond the liver that enables robust clinical efficacy remains to be addressed.

Given the versatility of RNA properties, innovative RNA-based technologies are intensively emerging. In addition to antisense and siRNAs, novel attractive classes of RNA therapeutics (e.g. saRNAs, oligonucleotide-based artificial ribonucleases and RNA-PROTAC) demonstrate highly encouraging outcomes to modulate gene and protein expression, opening a new repertoire of promising platforms for drug discovery. Oligonucleotide-based delivery systems such as pRNA, NA nanostructures and SNAs have been explored as well and may offer relevant platforms to deliver nucleic acids beyond hepatic cells. Even though more understanding of their mechanism and behaviours is needed, the emerging RNA-based technologies described in this review present unique therapeutic and delivery potentials and will establish a path towards expanding RNA-based applications.
